# Microglia-vascular interactions after spinal cord injury: regulatory mechanisms and therapeutic advances

**DOI:** 10.3389/fimmu.2026.1856481

**Published:** 2026-06-02

**Authors:** Yulin Zhao, Weiyun Wang, Shihao Li, Manglai Li, Wenwu Zhang, Tan Lu, Lei Wang, Tao Han, Wenjie Ren

**Affiliations:** 1 The Third Affiliated Hospital of Henan Medical University, Institutes of Health Central Plains, Henan Medical University, Xinxiang, Henan, China; 2 Tianjin Medical University, Tianjin, China; 3 Xinxiang Key Laboratory for Molecular Oncology, Henan Medical University, Xinxiang, Henan, China; 4 Department of Orthopedics, The First Affiliated Hospital of Henan Medical University, Xinxiang, Henan, China; 5 Clinical Medical Center of Tissue Engineering and Regeneration, Henan Medical University, Xinxiang, Henan, China

**Keywords:** angiogenesis, immunology, microglia, microglia-vascular interactions, spinal cord injury

## Abstract

The repair process following spinal cord injury (SCI) involves intricate crosstalk between neuroimmune and vascular systems, with microglia-vascular interactions increasingly recognized as an important regulatory interface that may shape both secondary injury progression and neural regeneration. This review delineates the dual role of angiogenesis in SCI: functionally mature neovessels can facilitate restoration of blood supply, provide neurotrophic support, and offer guidance cues for axonal regrowth; in contrast, structurally abnormal and hyperpermeable vessels can exacerbate blood-spinal cord barrier (BSCB) disruption, amplify inflammatory responses, and perpetuate local hypoxia, thereby impeding functional recovery. As the primary resident immune cells of the central nervous system (CNS), microglia substantially influence the initiation, extension, and maturation of angiogenesis through dynamic, context-dependent functional states that are often operationally discussed within the M1/M2 framework. Conversely, vascular injury, barrier leakage, hypoxia-associated signaling, and endothelial-derived mediators can reshape microglial activation and function, establishing a bidirectional interaction network. Therapeutic strategies targeting this axis are shifting from indiscriminate angiogenesis promotion toward multimodal and stage-aware interventions, including modulation of microglial states, exosome-mediated delivery of bioactive molecules, functionalized biomaterial scaffolds, pathway-directed pharmacological approaches, and integration of physical rehabilitation strategies. These approaches have shown encouraging effects in preclinical models, including improved vascular maturation, reduced inflammatory injury, and better neurological outcomes. Future studies should combine single-cell multi-omics, spatially resolved profiling, *in vivo* imaging, and smart biomaterials to clarify the spatiotemporal dynamics of distinct cellular subpopulations, strengthen the preclinical evidence base, and support more rigorous translation of microglia-vascular axis regulation for SCI repair.

## 1 Introduction

Spinal cord injury (SCI) represents a devastating CNS trauma, in which neurological dysfunction results not only from primary mechanical damage but also from progressive tissue destruction driven by a cascade of secondary injury events (1, 2). For decades, research has primarily focused on the inherent limitations of neuronal regenerative capacity (3). However, accumulating evidence indicates that neurological recovery after SCI is also strongly influenced by dynamic remodeling of the local lesion microenvironment, particularly the interplay between neuroimmune and vascular systems (4, 5).

Within this complex pathophysiological network, microglia-vascular interaction has emerged in recent years as a core regulatory hub, and has attracted considerable scientific attention (6, 7). As resident immune sentinels of the CNS, microglia exhibit highly plastic activation states that are often described, for conceptual clarity, along pro-inflammatory and repair-associated axes historically framed by the M1/M2 terminology (8–10). This dynamic polarization is not simply an immune response; rather, it profoundly influences angiogenesis initiation, extension, and maturation, as well as the restoration of BSCB integrity (11, 12). At the same time, the vascular system functions as more than a passive conduit for blood supply; it releases metabolites, cytokines, and hypoxic signals—such as hypoxia-inducible factor-1α (HIF-1α) (13, 14)—thereby establishing a precise bidirectional intercellular communication mechanism (15–17).

Angiogenesis after SCI has a complex dual role, and its biological effects reflect the balance between vascular quality and quantity (18). On the one hand, newly formed blood vessels that are functionally mature and structurally intact can reconstitute local blood circulation, provide physical guidance scaffolds for axonal regeneration, and secrete various neurotrophic factors—all of which play pivotal roles in neural repair (19–21). Conversely, pathological vascular networks characterized by structural abnormalities, increased permeability, and inadequate pericyte coverage can exacerbate BSCB disruption, inflammatory cell infiltration, vasogenic edema, and local hypoxia, ultimately forming a vicious cycle with glial scarring that hinders regeneration (22, 23). In this process, microglia act as key “regulators,” and their phenotypic imbalance is recognized as one of the core mechanisms underlying dysregulated angiogenesis and failed repair (24–26).

Therefore, regulating microglial phenotypic balance and optimizing angiogenesis are essential for promoting neural repair post-SCI. Further studies are needed to define the mechanisms by which microglia modulate angiogenesis to lay a theoretical foundation for the development of novel therapeutic strategies. Therefore, a detailed investigation of the cellular and molecular interaction mechanisms governing “microglia-vascular interactions” not only holds significant theoretical value but also provides a novel framework for the development of breakthrough therapeutic strategies. This review aims to systematically elaborate on the bidirectional regulatory network between microglia and the vascular system post-SCI and comprehensively analyze the core role of this axis in coordinating inflammation resolution, vascular remodeling, and neural regeneration. On this basis, this review summarizes innovative therapeutic strategies targeting this axis that have emerged in recent years—including modulation of microglial polarization, precise intervention using exosomes and other cell-derived carriers, construction of functionalized biomaterial scaffolds, targeting of key signaling pathways, and multimodal synergistic treatments combining physical therapies. By elucidating “microglia-vascular interaction” across spatial and temporal dimensions, this review seeks to advance SCI repair from traditional single-modality therapies to modern strategies characterized by multi-targeted, sequential, and precise regulation.

## 2 Biological significance and controversies of angiogenesis after SCI

### 2.1 Key roles of angiogenesis in secondary SCI

Angiogenesis is defined as the process by which new blood vessels form from pre-existing vascular networks through “sprouting” (27). Within hours to weeks after SCI, the angiogenic program is rapidly activated and plays multiple critical roles in secondary injury (28). The injury core and surrounding ischemic regions face severe hypoxia and an energy crisis (29, 30). The formation of new blood vessels represents a direct means of re-establishing local blood circulation and supplying surviving cells with essential nutrients such as oxygen and glucose—fundamental for maintaining tissue homeostasis (31). Notably, while angiogenesis restores nutritional supply, the immaturity of early-stage vessels may perpetuate barrier disruption (32, 33). In addition, BSCB breakdown during this process leads to peripheral immune cell infiltration and the influx of harmful substances, exacerbating inflammation and creating a toxic microenvironment; thus, restoring BSCB integrity can limit the scope of secondary damage (34, 35). This indicates that newly formed vascular structures not only serve transport functions but also contribute to barrier protection. Neovasculature associated with neural repair, particularly tip cell-guided vascular sprouts, can serve as a physical scaffold (36). Regenerating axons can grow directionally along these vessels, a phenomenon termed “vascular co-guidance” (37). Mechanistically, components of the vascular basement membrane, such as laminin and collagen, provide a favorable matrix for axonal growth and serve as scaffolds for neural regeneration (38, 39). However, throughout SCI repair, vascular and neural responses extend beyond neurons. Together with microglia, astrocytes, pericytes, and oligodendrocyte precursor cells, they form a functional unit—the neurovascular unit (NVU) (40, 41). Interactions between nascent blood vessels and these cell types have recently become an active area of investigation, particularly the bidirectional regulation between microglia and blood vessels.

### 2.2 Correlation between neovascularization and neurological functional recovery after SCI

Recent studies in diverse disease models have further revealed the active regulatory role of neovascularization in neural repair, beyond its traditional roles in structural support and nutrient supply (2). Because newly formed blood vessels can modulate the repair microenvironment, they precisely regulate immune responses and neural progenitor cell behaviors in a spatiotemporal manner, thereby more tightly coupling angiogenesis to neuroregeneration (42). In traumatic brain injury models, a novel tissue-repairing macrophage subtype induced by M-CSF, IL-6, and TGF-β has been identified, exhibiting significant pro-angiogenic and neuroprotective gene expression profiles, and their infiltration is temporally aligned with neurological recovery (43). In SCI animal models, researchers have observed that regions with active angiogenesis often colocalize with areas of axonal regeneration and sparse nerve fiber remyelination (44–46). Enhancing angiogenesis through pharmaceutical agents (e.g., statins, erythropoietin) or genetic approaches (e.g., VEGF overexpression) not only increases vascular density in the injured area but also accompanies significant improvements in motor function (47–50). Conversely, inhibiting angiogenesis exacerbates tissue damage and functional impairment. Collectively, these findings indicate a spatiotemporal coupling between angiogenesis and neuroregeneration; vascular endothelial cells provide physical support and actively secrete neurotrophic factors, cytokines, and chemokines that promote neuroregeneration. These factors can indirectly regulate neuronal survival, synaptic plasticity, and remyelination (51–54). For example, molecules such as netrin-1—a laminin-related glycoprotein secreted by endothelial cells during embryogenesis—can directly guide axonal growth direction, while the secretion of other neurotrophic factors supports neuronal survival (55, 56). This spatiotemporal coupling suggests that effective neovascularization may represent an important enabling component of functional recovery. Clinically, advances in imaging technologies (e.g., dynamic contrast-enhanced MRI) have enabled *in vivo* evaluation of vascular changes in SCI patients (57, 58). Extensive preclinical evidence supports a positive association between effective vascular reconstruction and neural repair, whereas direct clinical evidence remains limited (59, 60). Although direct evidence remains limited, studies have found that patients with better local blood flow restoration after injury often exhibit more favorable neurological outcomes, highlighting the positive significance of vascular reconstruction (61). Neovascularization is not merely a passive supporter of neural repair but an active participant in regulating the repair microenvironment and coupling angiogenesis with neuroregeneration. A deeper understanding of the multifaceted mechanisms of angiogenesis in neural repair will support the development of innovative vascular-targeted therapeutic strategies and provide new intervention approaches for the repair of CNS diseases such as SCI.

### 2.3 Pathological consequences of abnormal angiogenesis after SCI

However, not all newly formed vessels are beneficial. Angiogenesis after SCI is often dysregulated, leading to the formation of functionally immature and structurally abnormal vascular networks that induce a series of pathological consequences (62). For instance, vascular endothelial growth factor (VEGF) expressed post-SCI strongly promotes angiogenesis but frequently results in morphologically distorted neovessels with irregular lumens and inadequate pericyte coverage, resulting in vascular structural disorganization. These abnormally structured vessels exhibit high permeability and fail to form a fully functional BSCB, thereby exacerbating vasogenic edema and inflammatory cell infiltration. Leaky vessels serve as channels for persistent inflammatory cell infiltration into the injury core, forming a vicious cycle (63). At the same time, functionally abnormal endothelial cells can overexpress adhesion molecules, further promoting immune cell adhesion and transendothelial migration, thereby inducing chronic inflammation and impairing tissue repair (64). Due to these structural and functional defects, newly formed vessels often suffer from insufficient perfusion and ineffective arteriovenous shunt formation and therefore fail to alleviate tissue hypoxia. Sustained hypoxia further upregulates VEGF expression via HIF-1α, exacerbating abnormal angiogenesis and forming a pathological positive feedback loop (65). In addition, abnormal blood vessels and surrounding reactive astrocytes can collectively form physical and chemical barriers—such as glial scars—that impede neural conduction (66–68). Specifically, vascular-associated scar tissue contains axonal growth inhibitory molecules such as chondroitin sulfate proteoglycans (CSPGs), which act as both physical and chemical barriers to axonal regeneration (69–71). Therefore, during reconstruction of the neurovascular network, it is essential to assess whether newly formed blood vessels establish effective perfusion pathways and minimize the adverse effects of abnormal vessels—considerations equally important as focusing on neovascular reconstruction itself.

**Figure 1:**
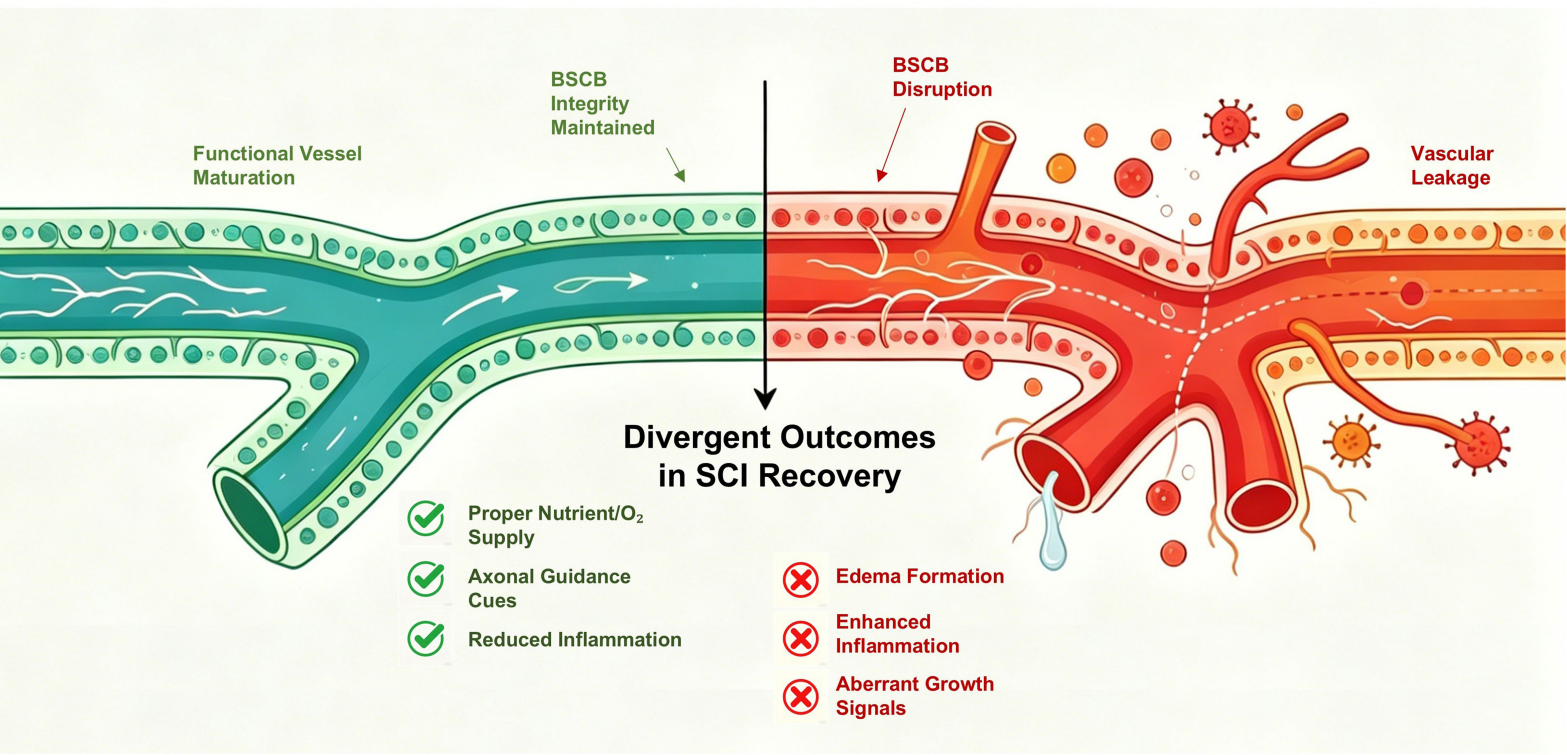
The schematic contrasts two pathways of neovascularization following SCI. (left) Functionally mature neovessels that maintain BSCB integrity, ensure nutrient/oxygen delivery, and provide axonal guidance cues; (right) abnormal leaky vessels that disrupt BSCB, exacerbate edema and inflammatory infiltration, and promote maladaptive axonal responses. This dual-outcome framework underscores the critical importance of promoting beneficial angiogenesis while limiting pathological vessel formation during SCI recovery.

Angiogenesis after SCI is a complex, dualistic process. The core controversy lies in the dialectical relationship between the quality and quantity of angiogenesis. Simply increasing vascular density while neglecting quality—such as maturity, integrity, and functionality—may be counterproductive. Current research has shifted from whether to promote angiogenesis to how to guide angiogenesis toward functional maturation and normalization. Understanding how different cell types (e.g., microglia, pericytes, astrocytes) collaborate to precisely construct a functional vascular microenvironment is crucial for developing next-generation SCI repair therapies. The dual-outcome framework of post-SCI neovascularization is schematically summarized in **Figure 1**.

## 3 Bidirectional regulation of microglia and vascular remodeling after SCI

**Figure 2:**
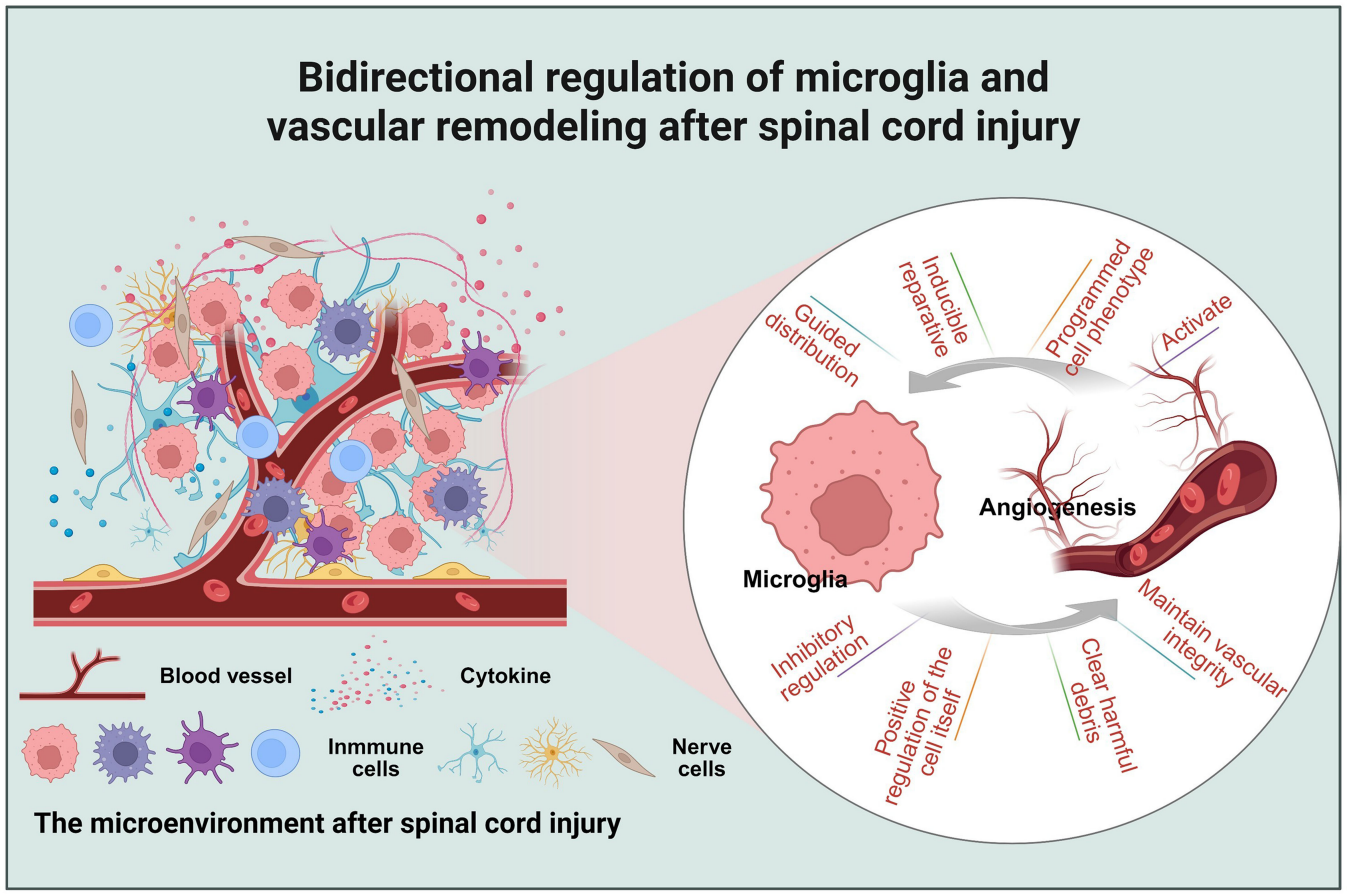
Schematic diagram of bidirectional regulation between microglia and vascular remodeling after SCI. After SCI, microglia and the vascular system form a bidirectional regulatory network. Microglia regulate angiogenesis and repair by changing their polarization states, thereby secreting factors such as VEGFA to promote new vessel formation. Meanwhile, the vascular system modulates microglial activation and function through hypoxic signals and metabolites, collectively influencing the reconstruction of the BSCB and neural repair.

The secondary injury cascade after SCI is a key contributor to poor neurological outcomes. Activated microglia do not constitute a homogeneous population; instead, they exhibit substantial state heterogeneity in response to microenvironmental signals. For readability, this review occasionally uses the conventional M1/M2 terminology as an operational framework, while recognizing that post-SCI microglial states *in vivo* are more complex and may not conform to a strict binary classification (72–74). High-throughput sequencing studies have further established that noncoding RNA expression networks are profoundly rewired after SCI, providing a previously unrecognized layer of regulation that governs microglial activation and their crosstalk with vascular cells (75). Historically, research on microglia (resident CNS immune sentinels) and the vascular system has followed relatively independent trajectories. However, recent studies indicate that these two systems form a tightly interconnected and interactive functional unit post-SCI. Microglia are far more than passive participants in inflammatory responses; they serve as central regulators of the microenvironment, with their activation states directly influencing angiogenesis efficiency and BSCB integrity (74, 76, 77). Therefore, the complex relationship between microglia and the vascular system post-SCI—termed microglia-vascular interaction—has become a major focus in SCI repair research (74, 78, 79). In-depth exploration of the mechanisms underlying this interaction is therefore crucial for identifying new therapeutic avenues for SCI repair. An overview of this bidirectional regulatory network is presented in **Figure 2**.

### 3.1 Inhibitory regulation of vascular sprouting by the temporal window of microglial polarization

The pathological microenvironment after SCI severely impedes beneficial angiogenesis (80, 81). As CNS immune sentinels, microglia undergo dynamic polarization after injury—particularly adopting specific activated states during the acute phase—which represents a central factor inhibiting angiogenic sprouting (82–84). The early post-injury inflammatory microenvironment is the primary barrier to vascular reconstruction. Within hours to days after injury, microglia rapidly adopt M1-like pro-inflammatory states and actively inhibit angiogenic sprouting and disrupt existing vascular networks through multiple mechanisms (85, 86). On the one hand, endothelial-monocyte activating polypeptide II (EMAP II)—a cytokine with potent anti-angiogenic activity—exhibits peak expression a few days after injury and directly inhibits vascular endothelial cell survival and tube formation, thereby suppressing early angiogenic sprouting attempts (87). Activated M1 microglia serve as the primary source of EMAP II after injury (88). Conversely, activated microglia are a major source of reactive oxygen species (ROS) after injury (89). Excessive oxidative stress not only directly damages vascular endothelial cells, inducing dysfunction and death, but also disrupts lipid metabolic processes essential for providing energy and building blocks for angiogenesis by inhibiting key metabolic enzymes such as fatty acid synthase (FASN) (90, 91). This metabolic disturbance further deteriorates the microenvironment required for angiogenic sprouting (92). Third, M1-like microglia can continuously secrete pro-inflammatory factors such as tumor necrosis factor-α (TNF-α) and interleukin-1β (IL-1β). These cytokines not only directly inhibit endothelial cell function—leading to increased BSCB permeability, edema, and local circulatory disturbances—but also collectively suppress effective angiogenic sprouting through both physical and chemical mechanisms (93, 94). Thus, although microglia are essential to tissue repair, excessive and uncontrolled M1 polarization during the acute phase represents a key obstacle to angiogenic sprouting. In summary, early after SCI, microglial polarization toward the M1 phenotype creates a temporal window that inhibits angiogenic sprouting through the release of anti-angiogenic factors, exacerbation of oxidative stress, and maintenance of inflammation. Targeted immunomodulation aimed at suppressing this detrimental polarization represents a key strategy for promoting spinal cord angiogenesis and endogenous repair. Studies have shown that mitochondrial transplantation derived from M2-like microglia can promote angiogenesis and inhibit M1 polarization, thereby facilitating functional recovery (95, 96). In parallel, ischemia, hypoxia, and plasma component extravasation resulting from vascular rupture constitute key initial signals driving microglial activation and phenotypic polarization (97). This interaction mechanism provides strong evidence for the development of effective strategies to treat CNS diseases and SCI.

### 3.2 Microglial promotion of angiogenesis and repair

Within the complex post-SCI microenvironment, neuroinflammation and vascular dysfunction are regarded as two core drivers of tissue damage expansion and impaired neural regeneration (98). Among these, the activation and polarization of microglia, together with the destruction and repair of the spinal vascular system, constitute central components of the pathophysiological process. The molecular features of these functional subsets may partially overlap with classical M2 markers, but their functional status *in vivo* is more complex (99). Zeng et al. demonstrated that acute-phase microglial depletion post-SCI—although it effectively limited the spread of pro-inflammatory responses—failed to promote angiogenesis; instead, it resulted in lesion area expansion, downregulation of angiogenesis-related factors, and ultimately impaired functional recovery. This highlights the importance of microglia in maintaining microenvironmental balance within a specific temporal window (100). Following SCI, disruption of local homeostasis activates resident microglia, which rapidly transform from a resting ramified morphology to an activated amoeboid form, initiate proliferation, and become among the earliest responders in the injury microenvironment. Several studies have shown that microglia serve as a major source of VEGFA secretion (101, 102). Deng et al. found that zinc ions can promote microglial VEGFA secretion, thereby stimulating endothelial cell proliferation, migration, and tube formation—effectively improving the post-injury hypoxic microenvironment (103). Using single-cell RNA sequencing, Yao et al. identified that microglia and macrophages specifically regulate certain endothelial cell subpopulations via the SPP1 and IGF signaling pathways, thereby precisely promoting angiogenesis (104). Beyond microglia themselves, microglia-derived exosomes represent important signal transduction carriers (105, 106). Peng et al. demonstrated that microglia-derived exosomes (MG-Exos) can activate the Keap1/Nrf2/HO-1 antioxidant signaling pathway, inhibit oxidative stress, protect endothelial cell survival, and enhance their tube formation and migration capabilities—significantly promoting vascular regeneration and neurological function improvement both *in vitro* and *in vivo* (107). In summary, microglia are activated early after SCI and actively participate in regulating vascular repair and regeneration through multiple mechanisms. Their functions include direct secretion of key factors such as VEGFA to improve the hypoxic microenvironment and promote endothelial cell function as well as precise regulation of specific endothelial cell subpopulations and guiding angiogenesis. In addition, microglia-derived exosomes can effectively protect endothelial cells and enhance their repair capabilities through antioxidant signaling pathways. Collectively, these findings establish the pivotal role of microglia in linking neuroinflammation with vascular dysfunction, highlighting them as a potential key target for simultaneously intervening in inflammatory responses and promoting vascular reconstruction to improve neural repair.

### 3.3 Microglial promotion of vascular remodeling through clearance of vessel-associated debris

Post-SCI, effective vascular remodeling depends not only on the precise regulation of pro-angiogenic signals but also—more importantly—on the timely clearance of cellular debris within the injury microenvironment (108). As the primary resident immune cells of the CNS, microglia are well positioned to contribute to this clearance process through their robust phagocytic capabilities, thereby helping establish a permissive physical microenvironment for vascular repair and reconstruction (109). Primary vascular damage caused by SCI leads to the death of vascular components such as endothelial cells and pericytes, generating substantial cellular debris and denatured matrix proteins (110). Failure to clear these residues results in the continuous release of harmful substances—damage-associated molecular patterns (DAMPs) (111). Compelling evidence indicates that microglia are rapidly activated and migrate to sites of vascular injury where they perform critical clearance functions. Early studies suggested that microglia appear before angiogenesis, actively accumulating around blood vessels—and their timing strongly suggests a role in clearance during vascularization (112, 113). More importantly, this function is not limited to the initial stages of vasculogenesis but extends throughout the entire vascular remodeling process. In the late post-injury phase, when nascent vascular networks require further functional refinement, microglia remain active participants. Studies have found that in regions undergoing vascular pruning and around developing cavities, activated microglia/macrophages express high levels of the anti-angiogenic factor endostatin (79, 91, 114). These findings suggest that microglia/macrophage-lineage cells may participate in vascular remodeling through both paracrine signaling and phagocytic clearance of ineffective or redundant vascular structures, thereby contributing to the maintenance of vascular network homeostasis. Consistent with a supportive role of microglia in vascular repair, pharmacological microglial depletion has been associated with lesion area expansion, impaired vascular reconstruction, and poorer functional recovery (92, 100). However, these outcomes likely reflect the combined loss of multiple microglial functions, including trophic support, immune regulation, and potentially debris clearance. Therefore, although impaired phagocytic activity may contribute to deterioration of the angiogenic microenvironment, its specific causal contribution requires further clarification. In addition, in the normal CNS, bone marrow-derived vasculature-associated myeloid cells expressing monocytic/microglial markers have been shown to phagocytose vascular components (115). Although these findings do not directly establish an SCI-specific microglial mechanism, they provide complementary evidence that myeloid-lineage cells can participate in vascular-associated clearance processes. In summary, available evidence suggests that microglial and macrophage-lineage phagocytic activities may contribute importantly to vascular remodeling after SCI, although their cell-type-specific roles require further clarification. Beyond classical paracrine signaling, clearance of vascular-associated debris may represent an important supportive mechanism for maintaining a repair-permissive microenvironment (81, 116). From creating space for angiogenic ingrowth to assisting the refinement of nascent vascular networks, these clearance-related functions provide a rationale for further investigating phagocytosis-oriented therapeutic strategies in SCI.

### 3.4 Microglial maintenance of vascular integrity

**Figure 3:**
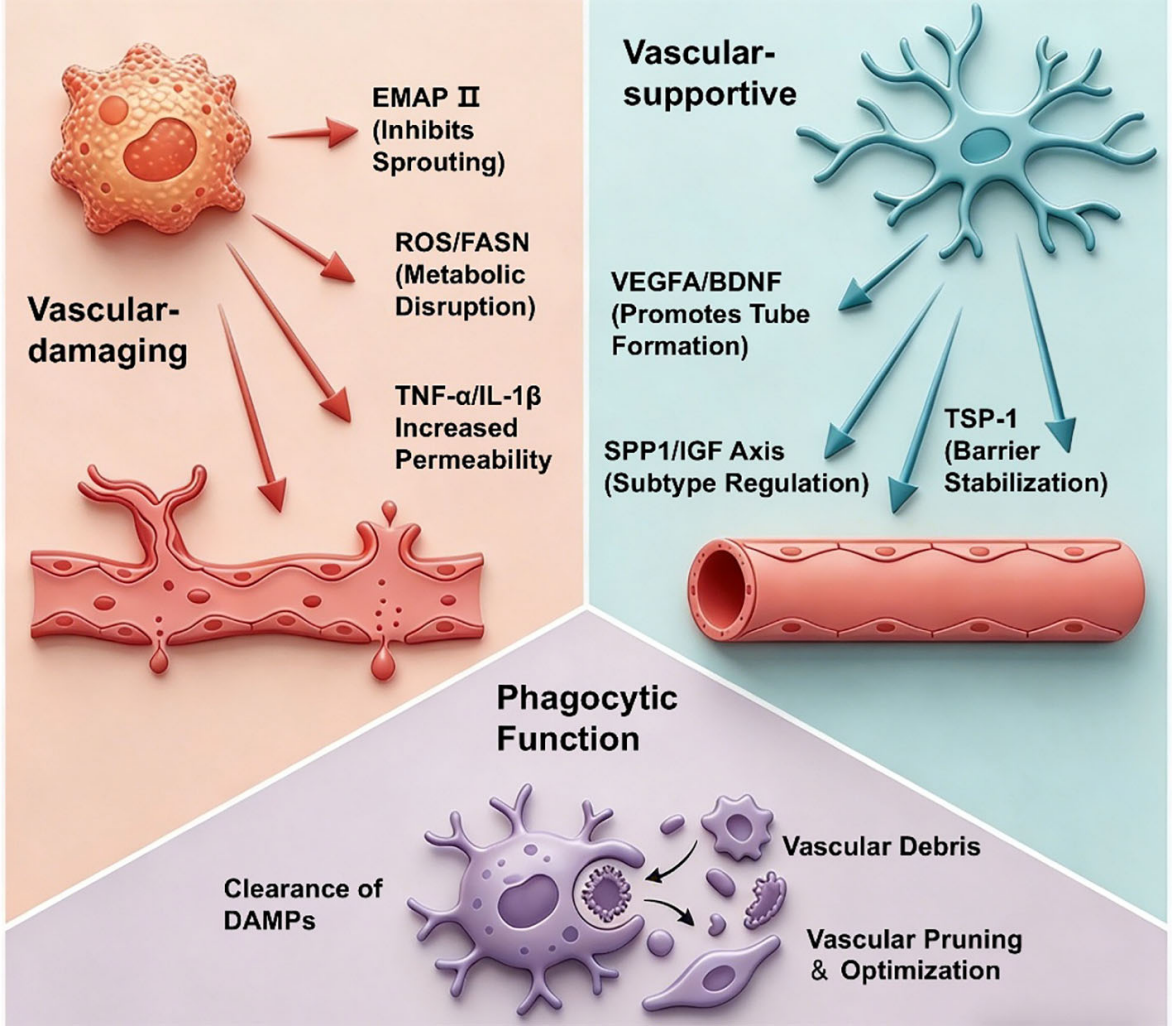
Multifaceted regulatory mechanisms of microglia in post-SCI angiogenesis and vascular remodeling. Pro-inflammatory-like microglial states disrupt the BSCB and suppress angiogenesis by secreting inflammatory cytokines (TNF-α, IL-1β, EMAP II) and inducing metabolic/oxidative stress (via ROS and FASN inhibition). Repair-associated microglial states promote functional angiogenesis and tube formation through VEGFA/BDNF signaling. In this schematic, amoeboid and ramified morphologies are used illustratively and should not be interpreted as one-to-one equivalents of specific functional states. This process is refined by SPP1/IGF-mediated endothelial subpopulation regulation and putative Tsp-1-associated vascular stabilization. Phagocytic microglia facilitate vascular network remodeling by clearing DAMPs and debris, highlighting their plastic role in orchestrating post-injury vascular repair.

After SCI, microglia actively participate in maintaining vascular integrity through multiple mechanisms and are essential for the stability of the NVU. Microglia accumulate around blood vessels and regulate vascular permeability and repair processes through phenotypic transformation (117). In retinal disease models, a specific microglial subset has been shown to maintain blood–retinal barrier stability via Tsp-1 (118). Notably, microglia also highly express Tsp-1 after SCI (119, 120), suggesting that this vascular-protective mechanism may be conserved in the spinal cord; however, direct functional validation remains required. These findings suggest that in the context of SCI, microglia may play a key role in maintaining vascular structural and functional integrity by regulating local inflammatory responses, secreting vascular protective factors, and promoting vascular maturation—and may provide therapeutic targets for post-SCI neural repair. In SCI-specific studies, Halder and Milner found that microglia specifically accumulate around leaky blood vessels, and pharmacological microglial depletion with PLX5622 significantly exacerbates hypoxia-induced BSCB disruption. This study directly demonstrates the vital vascular protective functions of microglia. Furthermore, this protective mechanism is weakened in aged individuals, suggesting that age-related neurocellular dysfunction may exacerbate vascular pathology (121). In summary, microglia serve as crucial guardians of vascular integrity post-SCI. By specifically recruiting to leaky vessels and utilizing mechanisms such as phenotypic transformation and secretion of vascular protective factors (e.g., Tsp-1), microglia actively maintain BSCB stability and inhibit abnormal angiogenesis. Direct functional evidence has shown that microglial loss significantly intensifies vascular leakage and damage, highlighting their indispensable protective function. Notably, this protective mechanism appears to decline with aging, suggesting that aging may exacerbate secondary vascular injury (122). Therefore, targeting and enhancing the vascular homeostatic regulatory capacity of microglia may represent a potential therapeutic strategy to mitigate vascular dysfunction and promote post-SCI neural repair. The multifaceted roles of microglia in post-SCI angiogenesis and vascular remodeling—ranging from barrier protection to repair promotion and debris clearance—are comprehensively summarized in **Figure 3**.

### 3.5 Vascular system regulation of microglia

**Figure 4:**
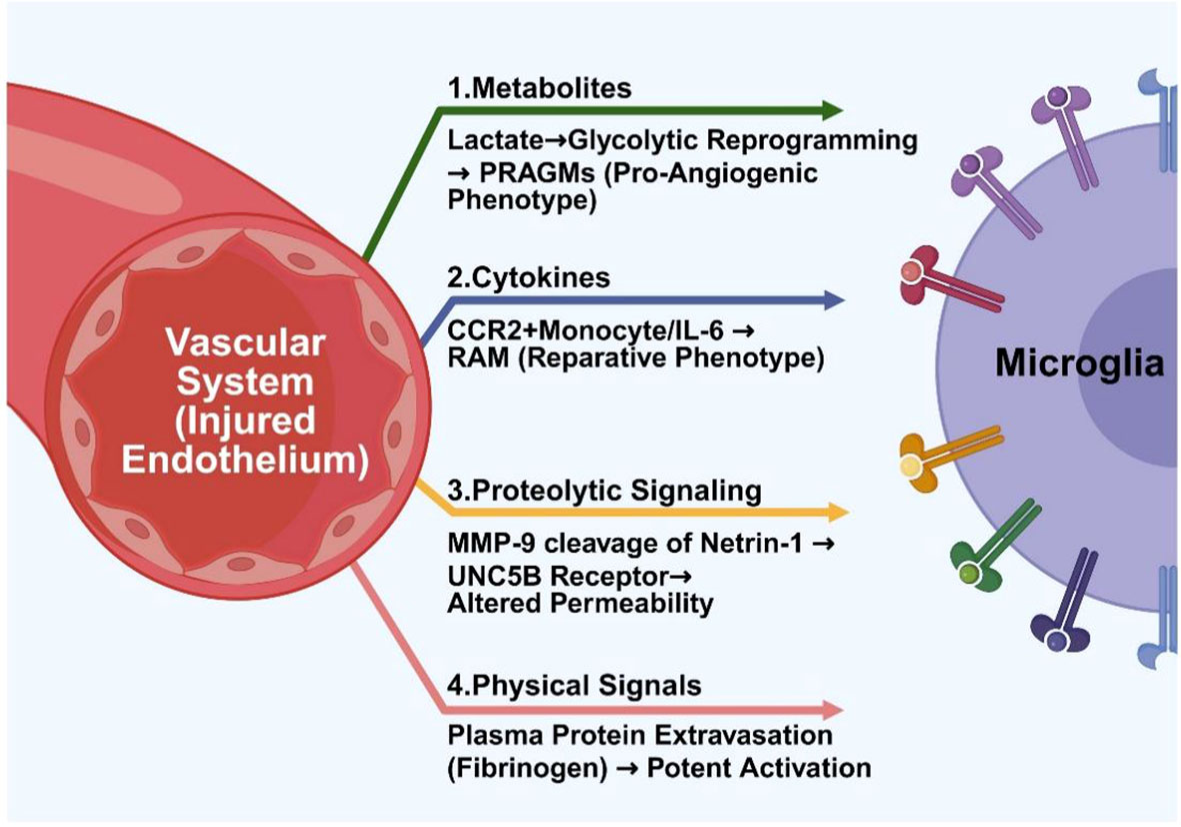
Vascular-to-microglial signaling pathways implicated in CNS injury and proposed to inform SCI repair. Endothelial-derived signals orchestrate microglial activation through four primary axes. 1. Metabolic: lactate-induced glycolytic reprogramming promotes a pro-angiogenic microglial (PRAGM) phenotype. 2. Cytokine: CCR2+ monocytes and IL-6 foster a reparative microglial (RAM) state with enhanced angiogenic activity. 3. Proteolytic: MMP-9-cleaved netrin-1 fragments (via UNC5B) modulate microglial function and vascular permeability. 4. Physical: extravasated plasma proteins (e.g., fibrinogen) act as potent triggers for microglial activation. These pathways establish the vasculature as a critical instructional hub for the neuroimmune microenvironment. Several axes are mainly supported by evidence from related CNS disease models and require SCI-specific validation.

**Figure 5:**
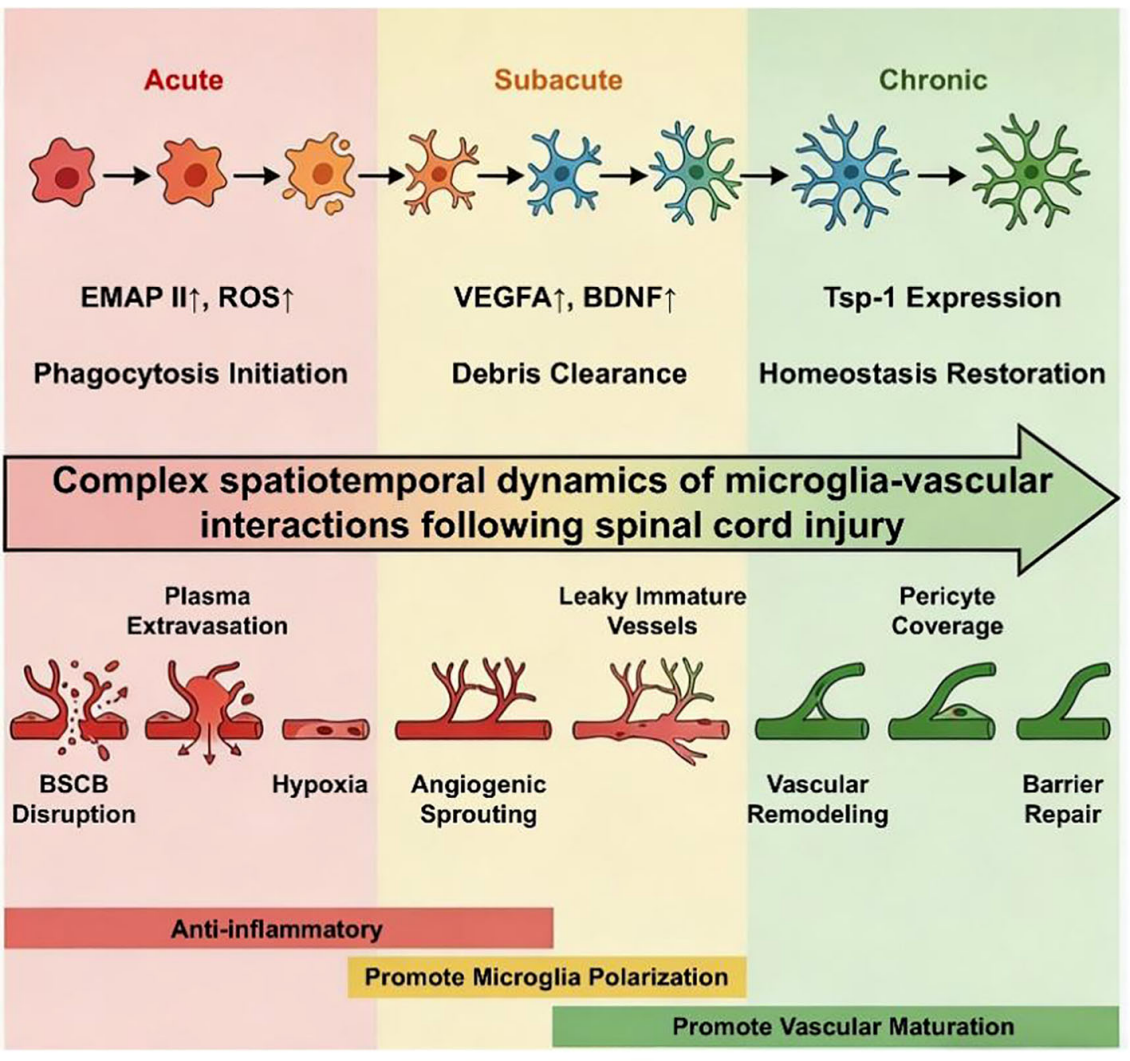
Spatiotemporal dynamics of microglia–vascular interactions and therapeutic windows after SCI. Upper track: a simplified schematic of microglial state dynamics, shifting from inflammation-associated programs (EMAP II, ROS) toward repair-associated and phagocytic programs (VEGFA, BDNF, Tsp-1) over time. Lower track: vascular response sequence, spanning initial BSCB disruption and hypoxia, angiogenic sprouting, and final barrier maturation. Bottom bars: proposed therapeutic windows: anti-inflammatory (acute), promotion of repair-associated microglial state transitions (subacute), vascular maturation (late subacute/chronic). This framework outlines a stage-specific strategy for modulating the microglia–vascular axis to optimize post-injury repair.

In the pathological microenvironment after SCI, the vascular system acts as a dominant signal source, releasing various specific molecules that instruct microglial activation and functional polarization (123). As illustrated in **Figure 4**, this instructive signaling occurs through four primary axes: metabolic, cytokine, proteolytic, and physical. Strong evidence for this regulatory pattern has been obtained in other CNS disease models. In pathological retinal models, lactate released by hypoxic vascular endothelial cells can drive microglial glycolytic reprogramming, polarizing them toward a unique pro-angiogenic phenotype (PRAGMs) (124); in cerebral hemorrhage models, CCR2+ monocytes derived from the vascular lumen are the primary source of IL-6, which—by binding to microglial IL-6R—directly drives their transformation into a reparative phenotype (RAM) with pro-angiogenic capabilities (76); in diabetic retinopathy, matrix metalloproteinase 9 (MMP-9) in the vascular microenvironment cleaves Netrin-1 to generate active fragments that directly regulate microglial function via the UNC5B receptor, exacerbating vascular leakage (125); additionally, HIF-1α-mediated hypoxic inflammatory responses in myeloid cells have been found to strictly depend on upstream activation of vascular endothelial cell IRE1α signaling (97). Together, these findings from related CNS disease models suggest that injured vasculature may influence microglial phenotypes and response patterns through multiple pathways—including metabolites, cytokines, protease modifications, and endoplasmic reticulum stress signals—establishing its core role as an upstream regulatory hub and providing a clear theoretical basis for remodeling the post-SCI neuroimmune microenvironment by targeting vasogenic signals. In SCI-related studies, BSCB disruption serves as a potent activation signal; leaky vessels allow plasma proteins such as fibrinogen and thrombin to enter the spinal cord parenchyma, which act as powerful microglial activators (126). Local ischemia and hypoxia caused by vascular damage induce the expression of transcription factors such as HIF-1α, which in turn upregulate VEGF, chemokines (e.g., CCL2), and other factors that continuously drive microglia/macrophage activation and recruitment (127). Furthermore, newly formed vascular sprouts provide physical tracks for microglial migration and spatial distribution, guiding them to converge in the injury core to perform their functions (112). The vascular system is not only a target of injury but also an active regulatory hub that may provide instructive cues shaping microglial activation, phenotype, and function after SCI (128). Thus, blood vessels can strongly activate microglia directly through leaked plasma proteins (e.g., fibrinogen) and program their response patterns via various molecular pathways—such as hypoxia-induced metabolic products (e.g., lactate), cytokines (e.g., IL-6), protease modification signals (e.g., MMP-9/Netrin-1) (129), and endoplasmic reticulum stress signals. At the same time, neovascularization provides a physical scaffold for microglial migration and spatial distribution. These findings from diverse CNS disease models provide an important conceptual framework and potential molecular targets for understanding how the vascular system can actively instruct microglia after SCI; however, their exact roles in the SCI microenvironment require further elucidation. Collectively, the spatiotemporal dynamics of these reciprocal interactions and the proposed stage-specific therapeutic windows are depicted in **Figure 5**.

## 4 Therapeutic strategies and mechanisms targeting microglia-vascular interactions after SCI

**Figure 6:**
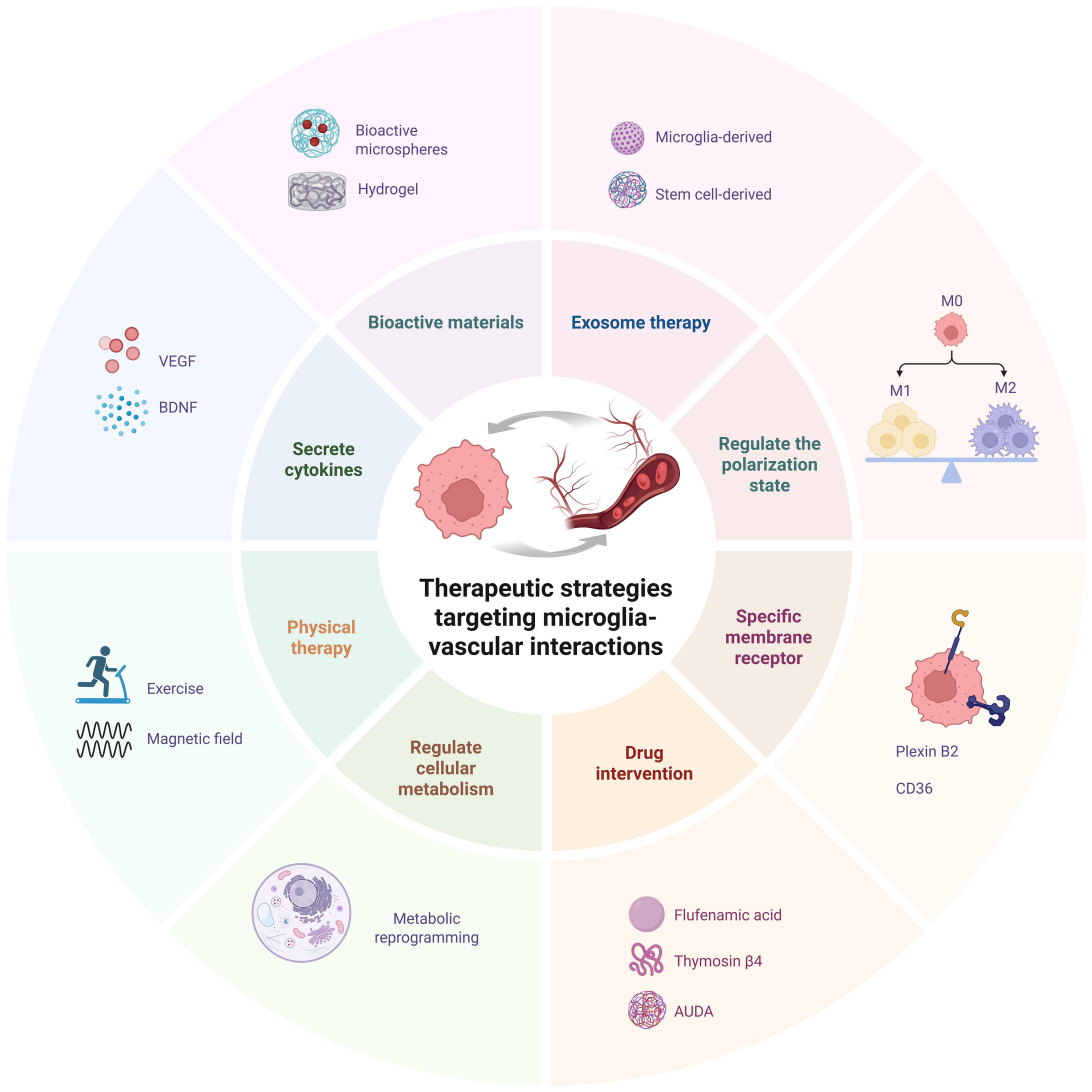
Schematic diagram of therapeutic strategies targeting microglia-vascular interactions after SCI. Current therapeutic strategies focus on precise regulation of microglia-vascular interactions. Key approaches include: promoting repair-associated microglial state transitions; utilizing exosomes for delivering active molecules; constructing functionalized biomaterial scaffolds to provide a local repair microenvironment; and targeting key receptors or metabolic pathways. These multimodal strategies aim to synergistically optimize angiogenesis, reduce inflammation, and promote neurological functional recovery.

In SCI and other CNS injuries, microglia—resident CNS immune cells—not only participate in neuroinflammatory responses but also play key roles in angiogenesis, BSCB integrity maintenance, and neural repair. In recent years, an increasing number of studies have focused on modulating microglial functional states to influence angiogenesis and repair, establishing the “microglia-vascular interaction” as a novel therapeutic target. This axis emphasizes the synergistic effects of the neuroimmune and vascular systems in injury repair, thereby providing a new theoretical framework for the development of multi-targeted therapeutic strategies. A schematic overview of these multimodal strategies targeting the microglia-vascular axis is provided in **Figure 6**.

### 4.1 Mechanistic targets in modulating microglia-vascular crosstalk

Understanding the cellular and molecular targets through which microglia and the vasculature communicate is essential for designing effective therapies. These targets can be broadly categorized into four areas: polarization state, secretory profile, membrane receptors, and metabolic programs.

#### 4.1.1 Microglial polarization states

The functional impact of microglia on the vasculature is highly dependent on dynamic phenotypic polarization. Pro-inflammatory-like microglial states release cytotoxic factors such as iNOS and TNF-α that damage endothelial cells, increase BSCB permeability, and inhibit angiogenic sprouting (92). In contrast, repair-associated microglial states secrete anti-inflammatory cytokines (e.g., IL-10, TGF-β) and upregulate markers such as Arg-1 and CD206, thereby creating a microenvironment that supports endothelial survival, angiogenic sprouting, and vascular maturation (130, 131). Key signaling pathways controlling this switch include NF-κB, which supports pro-inflammatory programs, and STAT3/STAT6, which favor repair-associated state transitions. The SIRT1/NF-κB axis has also been recognized as an important regulatory node in macrophage polarization (132). Estrogen receptor signaling, for instance, can suppress NF-κB activity and facilitate repair-associated transition (133). In addition, complement activation has been shown to recruit microglia and induce a repair-associated phenotype (134). Specific microRNAs, including miR-124 and miR-21, reprogram the microglial transcriptome toward a repair-associated transcriptional profile by downregulating pro-inflammatory gene networks (135). Conversely, sustained pro-inflammatory microglial programs reinforce vascular toxicity (132), and strategies that deplete or inhibit hyperactivated pro-inflammatory microglial states can alleviate vascular injury (92).

#### 4.1.2 Microglial secretion profiles

Microglia regulate angiogenesis directly through paracrine secretion of vasoactive factors. Following SCI, microglia become a major source of vascular endothelial growth factor A (VEGFA), which promotes endothelial cell proliferation, migration, and survival via VEGFR2-mediated signaling and the PI3K/AKT/Bcl-2 pathway (101–103). Microglial brain-derived neurotrophic factor (BDNF) acts on pericyte TrkB receptors to enhance pericyte coverage and vascular maturation (100). Single-cell transcriptomic analyses have further revealed that microglia communicate with specific endothelial subpopulations (e.g., Cdk1+ cells) through the osteopontin (SPP1)-CD44 axis and insulin-like growth factor (IGF) pathways, thereby driving directional reconstruction of the vascular network (104). The SDF-1α/CXCR4 axis represents another important regulatory node, capable of orchestrating both microglial recruitment and endothelial angiogenic responses (136). In addition, activated protein C (APC) exhibits dual protective effects: it preserves endothelial tight junctions (e.g., ZO-1) and suppresses microglial MMP-9 expression (137, 138).

#### 4.1.3 Microglial membrane receptors

Microglia sense microenvironmental cues through a repertoire of surface receptors that directly affect vascular functions. CD36, a scavenger receptor involved in lipid metabolism, modulates microglial activation and inflammatory responses (139). Inhibition of the CD36 pathway has been associated with reduced fibrosis and improved vascular microenvironment after SCI (131). Plexin B2 (Plxnb2), highly expressed on microglia during glial scar formation, may influence the structure of the scar-associated vascular network (140). The purinergic receptor P2Y12, a critical mediator of microglial motility and chemotaxis, also contributes to injury responses in the CNS (141). In other CNS models, triggering receptor expressed on myeloid cells 2 (TREM2) and the fractalkine receptor CX3CR1 have been shown to regulate microglial phagocytosis, inflammatory phenotype, and interactions with endothelial cells, thereby impacting angiogenesis and blood-brain barrier integrity (142, 143). Notably, LILRB2-mediated inhibition of TREM2 signaling provides an additional layer of regulatory control over microglial functions (144). These receptors represent precise molecular targets for steering microglia toward phenotypes that promote vascular maturation and barrier repair.

#### 4.1.4 Metabolic reprogramming of microglia

Microglial metabolic programs have emerged as central regulators of their vascular functions, often operating independently of classical M1/M2 surface markers (145). This concept of metabolic reprogramming as a driver of cellular adaptation extends across tissue types, with nutrient metabolism playing similarly critical roles in maintaining cell survival within avascular environments (146). The balance between glycolysis and oxidative phosphorylation (OXPHOS) dictates microglial secretory output: pro-inflammatory microglia rely on aerobic glycolysis (the Warburg effect), whereas reparative microglia utilize OXPHOS and fatty acid oxidation (FAO) to support vascular repair factor secretion (147–149). Key metabolic targets include the glycolytic enzymes HK2 and PFKFB3, the OXPHOS regulator PGC-1α, and the redox-sensitive transcription factor Nrf2 (150). Mitochondria are both energy factories and major sources of reactive oxygen species (ROS); excess mitochondrial ROS activates NF-κB and directly damages endothelial cells (61). The mTOR pathway integrates nutrient and energy signals, with mTORC1 hyperactivation driving pro-inflammatory polarization and angiogenesis inhibition, while AMPK activation shifts metabolism toward OXPHOS/FAO and vascular repair (151). Amino acid and lipid metabolism further contribute: glutamine serves as a TCA cycle fuel and precursor for the antioxidant glutathione, and fatty acid synthase (FASN) activity modulates inflammatory mediator synthesis (146, 152); conversely, blockade of glutamine synthetase has been shown to enhance the inflammatory response in microglial cells (153). The hypoxic SCI environment stabilizes HIF-1α, promoting glycolysis and lactate production; lactate, in turn, can act as a signaling molecule that, at sustained moderate levels, polarizes microglia toward a pro-angiogenic phenotype (PRAGMs) (124, 154). Collectively, these metabolic nodes offer an array of targets for reshaping microglial vascular regulation. This mechanistic framework also explains why metabolism-oriented interventions are discussed in Section 3.2.1 as an integral therapeutic extension of microglial metabolic reprogramming.

### 4.2 Therapeutic modalities targeting the microglia-vascular axis

The mechanistic targets described above have been exploited through a variety of therapeutic modalities, as detailed below.

#### 4.2.1 Pharmacological interventions

Pharmacological agents that directly target the molecular sites outlined in Section 3.1 have shown considerable promise. Flufenamic acid (FFA), a Trpm4 channel inhibitor, protects BSCB integrity by inhibiting the SUR1-TRPM4 complex and reducing MMP-9 expression (81). Thymosin β4 promotes endothelial migration and tube formation by activating integrin-linked kinase (ILK) while concurrently suppressing NF-κB-driven inflammation (155). The mitochondria-targeted antioxidant MitoQ neutralizes excess superoxide, restores microglial energy metabolism, and ensures proper VEGF secretion (61). Soluble epoxide hydrolase inhibitors (e.g., AUDA) elevate anti-inflammatory epoxyeicosatrienoic acids (EETs), thereby suppressing microglial activation and promoting angiogenesis (156). Targeting the mitochondrial DNA–STING–NF-κB axis with agents such as cryptotanshinone has also been shown to alleviate microglia-mediated inflammatory damage in ischemic retinopathy (157). Modulators of metabolic targets include PFKFB3 inhibitors that reverse the glycolytic bias of pro-inflammatory microglia (150), mTORC1 inhibitors such as low-dose rapamycin that normalize microglial metabolism and improve vascular density (151), and PPARγ agonists like pioglitazone that promote FAO-driven M2 polarization and vascular maturation (158). The glutamine synthetase activator valproic acid enhances microglial antioxidant defenses (153). Furthermore, protein-based agents such as SDF-1α, when delivered intrathecally, promote functional recovery by enhancing microglial and astrocytic reactivity and stimulating angiogenesis (136), while activated protein C (APC) simultaneously stabilizes the vasculature and suppresses microglial MMP-9 expression (137). Together, these pharmacological interventions illustrate the multi-target approach required to tackle the microglia-vascular axis. Thus, the metabolism-oriented strategies described here directly translate the mechanistic concept of microglial metabolic reprogramming outlined in Section 3.1.4 into therapeutic intervention.

#### 4.2.2 Exosome-based and cell therapies

Exosomes have emerged as natural nanoscale carriers capable of modulating the microglia-vascular axis with low immunogenicity (159). Microglia-derived exosomes (MG-Exos) have been shown to deliver signaling molecules that activate the Keap1/Nrf2/HO-1 antioxidant pathway in endothelial cells, scavenging ROS and enhancing endothelial survival and tube formation (107). Extracellular vesicles from human amniotic mesenchymal stem cells (hAMSC-Exos) are taken up by microglia, where they inhibit pro-inflammatory polarization via the Akt/STAT3 pathway; this phenotypic switch simultaneously releases the inhibition of pro-angiogenic factors such as VEGF, establishing a positive paracrine feedback loop between microglia and neovascularization (160). Umbilical cord mesenchymal stem cell-derived exosomes (ucMSC-exos), rich in miR-124 and miR-21, have been shown to systematically reprogram the microglial transcriptome toward a repair-associated phenotype, upregulating Arg-1 and CD206 and fostering a pro-angiogenic microenvironment (135). At the cellular level, transplantation of neural stem/progenitor cells derived from clinical-grade human iPSCs (hiPSC-NS/PCs) enhances angiogenesis and axonal regeneration while reducing demyelination and astrogliosis in non-human primate SCI models, effects that are likely mediated in part by modulation of host microglial activity (161). Combined strategies that co-deliver neural stem cells with exosomes further amplify these benefits by simultaneously replacing damaged cells and rapidly improving the lesion microenvironment (135).

#### 4.2.3 Biomaterial-based scaffolds and delivery systems

Tissue engineering strategies have evolved beyond providing mere physical support; through the integration of materials science, immunology, and regenerative medicine, they now serve as intelligent platforms for spatiotemporally precise regulation of microglia-vascular interactions. The importance of such dynamic microenvironmental control is underscored by the complex cellular interactions observed across diverse regenerating tissues (162). The key design principles and representative examples are outlined below. To clarify this subsection, the discussion is organized around five design dimensions: localized immunomodulatory release; exosome and gene delivery; vascular-normalizing scaffolds; matrix-guided microglial/endothelial organization; and responsiveness to inflammation, hypoxia, or ROS.

Localized immunomodulatory release. A foundational principle involves the sustained, local delivery of immunomodulatory agents to shift microglia from pro-inflammatory-like states toward repair-associated states without systemic side effects. This is typically achieved by incorporating bioactive ions, small-molecule drugs, or peptides into hydrogel or microparticle systems that gradually release their cargo at the injury site. For example, chitosan-modified zinc-containing bioactive glass microspheres (CS-MG@Zn/BGs) leverage sustained Zn^2+^ release to inhibit microglial NF-κB nuclear translocation, thereby promoting repair-associated microglial state transitions while simultaneously promoting VEGF secretion (19). Similarly, estrogen-loaded nanoparticles (SNP-E2) provide sustained release of estrogen, which activates microglial nuclear receptors to suppress NF-κB signaling and promote repair-associated polarization (133). α-galactose nanoparticles (α-gal NPs) exploit complement activation to recruit and polarize microglia toward repair-associated functional states *in situ* (134).

Exosome and gene delivery. Biomaterial scaffolds serve as ideal vehicles for delivering exosomes or gene-silencing constructs that reprogram microglial behavior. The scaffold protects the cargo from rapid degradation, prolongs its residence time, and enables controlled release kinetics. One representative example is the silk fibroin hydrogel loaded with Tim3 siRNA exosomes (RNAi-Tim3-Exo@SF), which combines siRNA-mediated gene silencing, exosome delivery, and hydrogel-based sustained release to knock down the immune checkpoint molecule Tim3 on microglia, effectively reversing their immunosuppressive and pro-inflammatory state and improving the lesion microenvironment for vascular reconstruction (163). Hydrogel-based microRNA delivery systems have also shown considerable promise for targeting inflammatory diseases (164).

Vascular-normalizing scaffolds. Beyond immunomodulation, rationally designed scaffolds can directly promote the formation of structurally and functionally normalized blood vessels—rather than the leaky, pathological vasculature that otherwise predominates after SCI. Such scaffolds achieve this by providing a compliant matrix that supports pericyte recruitment and endothelial barrier maturation, or by co-delivering factors that simultaneously stimulate angiogenesis while stabilizing nascent vessels (e.g., combined delivery of VEGF with angiopoietin-1 or PDGF-BB to enhance pericyte coverage). The dual-phase SilMA hydrogel system, which temporally separates an initial anti-inflammatory phase from a subsequent pro-angiogenic phase, illustrates how sequential release kinetics can steer vascular repair toward functional maturation (165). Porous SilMA scaffolds carrying dual-sensitive paclitaxel nanoparticles have similarly been designed to promote neuronal differentiation alongside vascular support (166).

Matrix-guided microglial/endothelial organization. The physical architecture and biochemical composition of the scaffold matrix can directly guide the spatial organization and functional polarization of both microglia and endothelial cells. Aligned fibrous or channeled scaffolds provide contact guidance that directs microglial migration, endothelial tube formation, and even axonal extension along defined axes, mimicking the natural neurovascular architecture. The inclusion of basement membrane components (e.g., laminin, collagen IV) or their bioactive peptide derivatives (e.g., RGD, YIGSR) provides substrate cues that promote endothelial monolayer formation and barrier function. Peptide-functionalized hydrogel microspheres have also been developed to remodel the vascular microenvironment through localized immuno-training (167). Three-dimensional gelatin sponge scaffolds, for example, furnish a porous architecture that facilitates cellular infiltration, vascular ingrowth, and degradation products that indirectly modulate perivascular microglial activity (168).

Responsiveness to inflammation, hypoxia, or ROS. Advanced scaffold designs incorporate environmentally responsive elements that sense and adapt to the pathological milieu of the injured spinal cord. ROS-responsive hydrogels contain oxidation-labile linkages (e.g., phenylboronate ester bonds, thioether groups) that selectively degrade upon exposure to elevated ROS levels at the injury site, releasing encapsulated therapeutic agents precisely when and where oxidative stress is maximal. One study developed a ROS-scavenging peptide hydrogel incorporating MnO_2_ nanoparticles that simultaneously eliminates excess ROS and delivers mesenchymal stem cells, thereby attenuating microglial oxidative damage while promoting angiogenesis and motor recovery after SCI (169). Hypoxia-responsive scaffolds exploit hypoxia-inducible systems to trigger the release of pro-angiogenic factors such as VEGF under low-oxygen conditions, amplifying neovascularization specifically in ischemic regions. Hypoxia-driven osteopontin has been identified as a key modulator of HIF1α-mediated VEGF-dependent angiogenesis, providing a molecular rationale for targeting this axis within hypoxic niches (170). Collectively, these stimuli-responsive systems embody the principle of “on-demand” therapy, where therapeutic intervention is automatically matched to the spatiotemporal evolution of secondary injury. Nanoparticle-based drug delivery platforms have been widely explored for ischemia-reperfusion injuries across various organ systems, further informing their application in SCI (171).

#### 4.2.4 Physical therapy and rehabilitation

In SCI rehabilitation strategies, existing studies have systematically analyzed post-SCI neural cell pathological changes and emphasized the key role of exercise training in promoting neural cell repair and regeneration (172). Exercise training activates intrinsic neuronal repair mechanisms by remodeling neuronal growth cones and cytoskeletal structures, regulating transcription factor activity, modulating protein signaling pathways, and influencing epigenetic modifications (173, 174). At the same time, it can regulate astrocyte activation states, optimize inflammatory responses and metabolic processes, promote astrocyte polarization, enhance angiogenesis, reduce glial scar formation, and regulate neurotrophic factor expression levels (175). In addition, exercise training effectively modulates microglial activation, promotes axonal regeneration, and improves phagocytic function—thereby optimizing the neural repair microenvironment. Gene expression analysis further showed that treadmill exercise training (TMT) can regulate molecular and cellular changes in the lumbar spinal cord post-thoracic SCI, restore injury-downregulated gene expression, and increase the expression of genes related to neural plasticity and angiogenesis—indicating that exercise training improves motor function recovery by promoting neurovascular remodeling (28). These findings highlight the potential of physical therapy as a non-invasive intervention in SCI rehabilitation, providing a theoretical basis and practical direction for clinical rehabilitation therapy.

Beyond active exercise training, passively applied physical factors have also been shown to promote SCI repair by regulating microglia-vascular interactions. Dey et al. found that exposure to extremely low-frequency magnetic fields can effectively improve motor function in rats with complete spinal cord transection (176). The protective mechanism is closely related to the regulation of microglial activity and iron metabolism: magnetic field treatment significantly reduced microglia/macrophage infiltration and collagen deposition at the injury site, and lowered iron levels accumulated due to hemorrhage. At the same time, magnetic field exposure promoted vascular endothelial growth factor expression around the lesion, enhancing angiogenesis (176). This study reveals a new mechanism for physical factor regulation of the repair process—namely, that iron ions released from post-SCI hemorrhage can exacerbate oxidative stress and activate microglia, forming a destructive cycle. Magnetic field intervention may break this vicious cycle by affecting intracellular iron metabolism or directly regulating microglial activation states—thereby alleviating inflammation and oxidative damage and creating a favorable microenvironment for angiogenesis. This provides experimental evidence for the development of non-invasive, drug-free physical therapy strategies.

These multimodal therapeutic strategies are summarized in **Figure 6**. The central principle is spatiotemporally precise regulation of microglial functional phenotypes, thereby optimizing the post-injury vascular microenvironment and ultimately providing the necessary conditions for neural repair and functional reconstruction. Current research has advanced from mere phenomenological descriptions to in-depth exploration of specific cellular subpopulations, molecular mechanisms, and the development of novel tools. Future studies should further utilize technologies such as single-cell spatial transcriptomics and live-cell imaging to precisely elucidate the dynamic roles of different microglial subpopulations at various stages of vascular reconstruction. Meanwhile, efforts should focus on developing conditional, controllable smart delivery systems—aiming to achieve real-time, precise interventions in microglial function and accelerate the translation of these promising strategies from basic research to clinical therapy.

## 5 Conclusions

The pathological process after SCI is highly complex, with the interplay between angiogenesis and the neuroimmune microenvironment serving as a core factor influencing injury repair outcomes. This review systematically summarizes the dual role of angiogenesis in SCI: functionally mature neovessels can reconstitute local blood circulation, provide structural scaffolds for neural regeneration, and offer neurotrophic support—serving as an important foundation for neurological functional recovery; conversely, abnormal and highly permeable pathological vasculature exacerbates BSCB disruption, inflammatory cell infiltration, and hypoxic conditions—forming a vicious cycle that impedes repair. At the same time, microglia, as CNS immune surveillance cells, regulate angiogenesis initiation, extension, and maturation through dynamic and context-dependent functional state transitions, thereby forming a bidirectional “microglia-vascular interaction.” Dysregulation of this axis is one of the key mechanisms underlying disordered angiogenesis and failed repair.

Current research has shifted from simply increasing vascular quantity to emphasizing vascular structural and functional maturity. Several strategies have been identified to optimize the post-injury vascular microenvironment—including modulation of microglial polarization, utilization of exosome-mediated intercellular communication, construction of functionalized biomaterial scaffolds, and targeting of key signaling molecules. These interventions have demonstrated potential in promoting neural regeneration and functional recovery in preclinical models. However, several scientific challenges remain—such as how to precisely regulate microglial state transitions in a spatiotemporal manner to guide functional vascular network formation, how to identify and target the specific roles of different endothelial cell subpopulations during repair, and how to achieve coordinated integration of immune modulation, vascular reconstruction, and neural regeneration under complex pathological conditions.

Future studies should further integrate advanced technologies such as single-cell spatial transcriptomics, live-cell dynamic imaging, and multi-omics integrated analysis to systematically reveal the molecular characteristics and interaction networks of different cellular subpopulations at various post-injury stages. At the same time, efforts should be devoted to developing intelligently responsive biomaterials, conditional gene regulatory systems, and targeted delivery platforms to achieve precise and dynamic intervention in microglia-vascular interaction—an important direction for promoting the translation of SCI repair strategies from basic research to clinical application. Under the framework of multidisciplinary collaboration, in-depth analysis of the molecular basis and regulatory mechanisms underlying the neuro-immune-vascular triad will provide SCI patients with clinically meaningful regenerative treatment strategies.
